# BARX1 promotes osteosarcoma cell proliferation and invasion by regulating HSPA6 expression

**DOI:** 10.1186/s13018-023-03690-z

**Published:** 2023-03-16

**Authors:** Xing Huang, Zhenhua Wang, Jing Zhang, Xiangzhi Ni, Guangjian Bai, Jiashi Cao, Chunlei Zhang, Zhitao Han, Tielong Liu

**Affiliations:** 1 Department of Orthopaedic Oncology, The Second Affiliated Hospital of Naval Medical University, No. 415 Fengyang Road, Huangpu District, Shanghai, 200003 China; 2 Department of Laboratory Medicine, Changzheng Hospital, Naval Medical University, No. 415 Fengyang Road, Huangpu District, Shanghai, 200003 China; 3 Department of Orthopedics, No. 455 Hospital of Chinese People’s Liberation Army, The Navy Medical University, No. 338 Huaihai West Road, Shanghai, 200052 China; 4 Department of Orthopedics, Nanjing Hospital of Traditional Chinese Medicine Affiliated to Nanjing University of Chinese Medicine, Nanjing, China; 5 School of Integrated Chinese and Western Medicine, Nanjing University of Chinese Medicine, Nanjing, China

**Keywords:** Osteosarcoma, BARX1, HSPA6, Proliferation, Invasion

## Abstract

Osteosarcoma (OS) is a bone tumour affecting adolescents. Dysregulation of Barx homeobox 1 (BARX1) expression is involved in various cancers, but its function and mechanism in the process of OS are undefined. This study revealed that BARX1 expression is higher in OS tissue than in adjacent normal tissue. Downregulation of BARX1 in OS cells significantly suppressed their proliferation and migration, whereas enforced expression of exogenous BARX1 exerted the opposite effects on OS cells. Subsequently, heat shock 70-kDa protein 6 (HSPA6) expression was clearly increased after BARX1 overexpression in OS cells, as confirmed by RNA sequencing. The dual-luciferase reporter assay confirmed that HSPA6 expression is directly regulated by BARX1. The in vitro assay indicated that silencing HSPA6 expression attenuated OS proliferation and migration induced by BARX1. A dual immunofluorescence labelling assay provided further evidence that BARX1 was overexpressed and associated with HSPA6 overexpression in OS tumour tissue. In conclusion, BARX1 promotes OS cell proliferation and migration by inducing the expression of HSPA6, which plays an oncogenic role in OS. BARX1 and HSPA6 can potentially act as novel therapeutic targets for OS.

**Supplementary Information:**

The online version contains supplementary material available at 10.1186/s13018-023-03690-z.

## Introduction

Osteosarcoma (OS) is a prevalent primary bone tumour mainly occurring in adolescents less than 20 years old [1]. OS most frequently occurs in the long extremity bone metaphysis. The currently reported 5-year overall survival rate of localized OS is 60–70%[2]. However, OS metastasis decreases the survival rate; the 5-year survival rate for individuals with lung metastases is 20–30% [3, 4]. Therefore, there is an urgent need to clarify the pathological mechanisms underlying OS metastasis to improve the survival of patients with metastatic OS.

Barx homeobox 1 (BARX1), belonging to the Barx homeobox protein family, has been investigated in multiple developmental contexts, including cranium, face, stomach, and muscle development [5–7]. BARX1 regulates the proliferation and metastasis of many tumours. Evidence has demonstrated that BARX1 promotes the proliferation of renal cell carcinoma and oesophageal adenocarcinoma [8, 9]. However, enforced BARX1 expression in gastric cancer can inhibit tumour proliferation. In endometrial carcinoma, silencing of BARX1 suppresses ERK/MEK signalling pathway activity, which decreases migration and cell viability [10]. BARX1 is considered a promising oncogenic transcription factor in non-small cell lung cancer (NSCLC) [11]. Notably, BARX1 expression is decreased in hepatocellular carcinoma (HCC), and its absence causes enhancement of tumour invasion and metastasis by inducing MGAT5 and MMP9 expression [12]. However, the function of BARX1 in OS is still unknown.

We aimed to clarify the role of BARX1 in OS. We detected BARX1 expression in tissues of OS patients. BARX1 was found to be significantly overexpressed in OS tumour tissue compared with adjacent normal tissue. In addition, our functional experiment demonstrated that silencing BARX1 in OS cells inhibited cell proliferation and invasion. We also identified the genes targeted by BARX1 via RNA sequencing (RNA-seq) and luciferase reporter assays. Above all, our study demonstrated that BARX1 plays an essential role in OS progression by regulating transcription and may be a therapeutic target.

## Materials and methods

### Human samples

Tumour tissues and normal adjacent tissues were obtained from OS patients from Shanghai Changzheng Hospital (Shanghai, China). All tissue samples were confirmed by histopathological biopsy. All participating patients were aware of the study and signed an informed consent form. Experiments were approved by the Naval Medical University Ethics Committee of Biomedicine and were performed following the Declaration of Helsinki.

### Immunofluorescence (IF)

The tissue slices were treated with EDTA buffer for antigen repair and subsequently blocked with 3% BSA. Anti-BARX1 (ab220859, Abcam) and anti-HSPA6 (13616-1-AP, Proteintech) primary antibodies were applied dropwise to the sections, and they were then incubated at 4 °C overnight, followed by treatment with the matching secondary antibody and incubation for 1 h at normal temperature. Immunofluorescence was detected by a three-colour fluorescence kit (Shanghai Recordbio Biological Technology, Shanghai, China). Then, DAPI was used to stain the nucleus. The images were observed and collected under an inverted microscope. The nucleus is dyed blue by DAPI, HSPA6 is coloured orange, and BARX1 is coloured green.

### Real-time PCR (RT-PCR)

RNA extraction from the tissue samples was performed by homogenization. Briefly, tissue samples were cut into fragments and transferred into 2 ml tubes, followed by the addition of TRIzol (1 ml) to each tube. The samples were then subjected to homogenization, and the supernatant was collected. The following steps were performed according to the instructions for TRIzol (Invitrogen Corporation, 15,596–018). We utilized Evo M-MLV RT Premix for qPCR and the SYBR Green Premix Pro Taq HS qPCR kit; reverse transcription and RT-PCR were performed using GAPDH as an internal control. All qPCRs were conducted on a 7900HT Fast Real-Time PCR System. Table 1 lists the primers used.

**Table 1:** RT-PCR primer sequences

Gene	Forward Primer (5′-3′)	Reverse Primer (5′-3′)
DNAJB1	AAGGCATGGACATTGATGACC	GGCCAAAGTTCACGTTGGT
HSPB1	ACGGTCAAGACCAAGGATGG	AGCGTGTATTTCCGCGTGA
BARX1	TTCCACGCCGGACAGAATAGA	AGTAAGCTGCTCGCTCGTTG
HSPA6	CAAGGTGCGCGTATGCTAC	GCTCATTGATGATCCGCAACAC
SERPINH1	TCAGTGAGCTTCGCTGATGAC	CATGGCGTTGACTAGCAGGG
CLDN6	TGTTCGGCTTGCTGGTCTAC	CGGGGATTAGCGTCAGGAC
ARC	AGCGGGACCTGTACCAGAC	GCAGGAAACGCTTGAGCTTG
GAPDH	CTGGGCTACACTGAGCACC	AAGTGGTCGTTGAGGGCAATG

### Western blotting (WB)

The protein content of the tissue samples was extracted using radioimmunoprecipitation assay buffer (RIPA) containing a phosphatase inhibitor, and the protein concentration was determined with a BCA kit. By using 10–12% polyacrylamide gels, the proteins were separated and then transferred to PVDF membranes, which were incubated with the primary antibodies BARX1 and HSPA6 at 4 °C for a whole night after being blocked with 5% skim milk, followed by adding the corresponding horseradish peroxidase-conjugated secondary antibodies.

### Cell culture and transfection

The OS cell lines and bone marrow mesenchymal stem cells (BMSCs) were procured from the Shanghai Cell Bank Type Culture Collection Committee (Shanghai, China). BMSCs and MG63, U2OS, HOS, and Saos-2 cells were cultured in DMEM with 10% foetal bovine serum (FBS) and 1% penicillin–streptomycin supplements at 37 °C and 5% CO_2_ in a humidified atmosphere. Plasmids and siRNAs were purchased from Shanghai GeneChem (Shanghai, China). A density of 2 × 10^5^ cells/well was used for culturing OS cells. Plasmid and siRNA cell transfections were performed following the Lipofectamine™ 2000 reagent protocols.

### Cell counting Kit-8 (CCK-8) assay

After seeding cells in 96-well plates, plasmids or siRNA were transfected into the cells. After 72 h of culture, a CCK-8 assay was utilized to evaluate cell viability per the protocols.

### Invasion assay

In a 24-well plate, the cell invasion assay was carried out. When the cell density reached 80–90%, the cells were resuspended and injected at a 1 × 10^5^ cells/millilitre into the Matrigel-coated upper layer of the Transwell chamber (Scipu002874; Corning Inc.), while for the chamber bottom layer, serum-containing medium was added. The cells were fixed with 4% formaldehyde and stained with 0.1% crystal violet after a 24-h culture period (C0121; Beyotime).

### Luciferase reporter assay

OS cells were transfected with HSPA6-luciferase reporter gene constructs for 48 h by Lipofectamine 2000 per the protocols, followed by OE-BARX1 treatment. Luciferase activity was measured by using a luciferase assay kit.

### RNA-seq

TRIzol Reagent (Invitrogen, USA) was applied to extract total RNA from MG63 cells (transfected with pcDNA-BARX1 or vectors for 24 h), followed by Illumina mRNA deep sequencing. The sequence results were obtained for each transcript as fragments per kilobase of exon per million reads.

### Statistics

All values are reported as the mean ± standard deviation (SD). Student’s t test was used to assess significant differences between group; the Kaplan‒Meier method and log-rank test were used to compare survival curves and determine statistical significance, respectively. Counting data were analysed with a χ^2^ test. *p* < 0.05 indicates a significant difference.

## Results

### BARX1 expression is upregulated in OS and correlated with OS progression

**Fig. 1:**
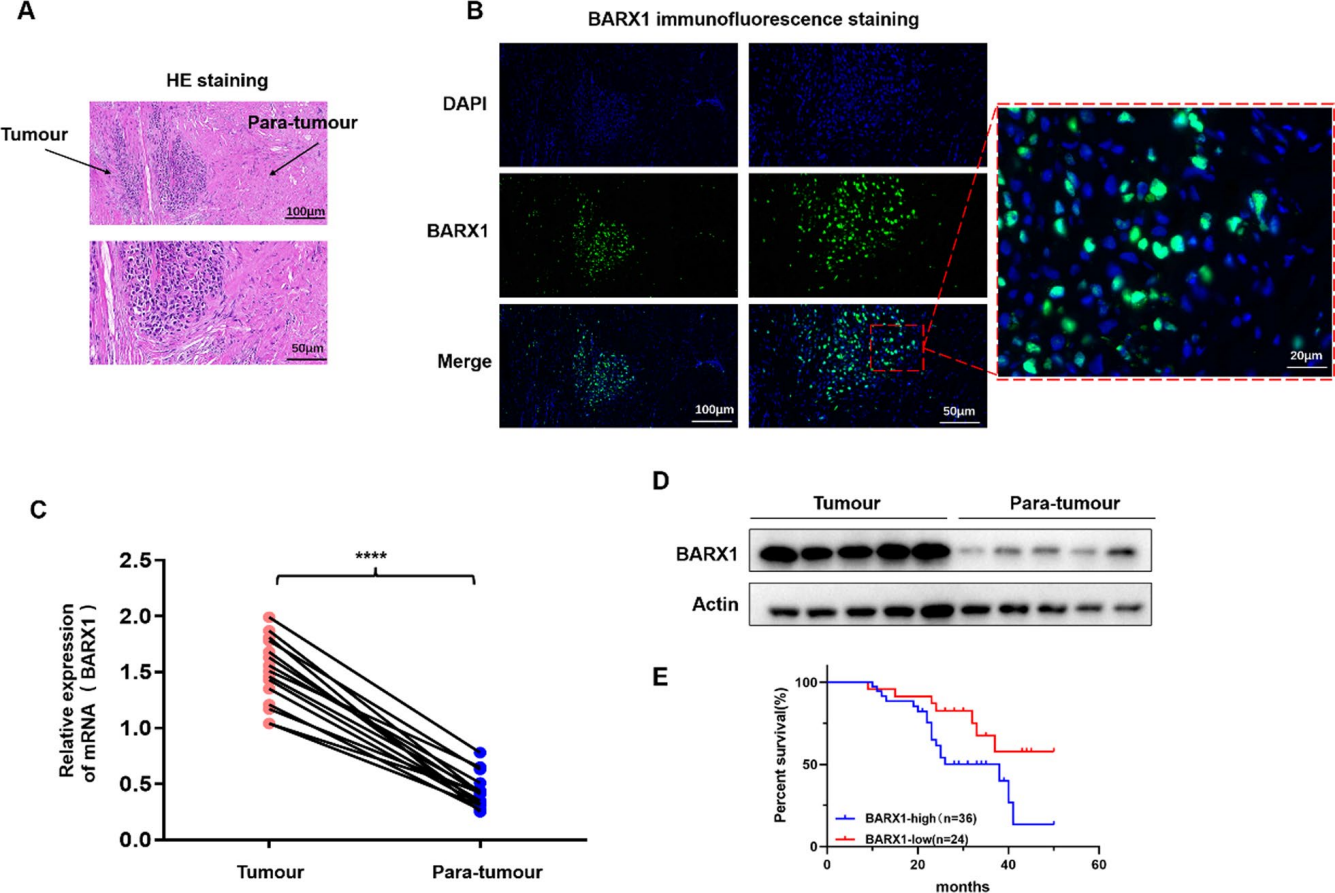
Barx homeobox 1 (BARX1) expression is upregulated in OS. **A** HE staining of tissues from OS patients. BARX1 **B** Immunofluorescence, **C** qRT-PCR, **D** Western blot analyses in tumour and adjacent tissues. **E** Kaplan‒Meier overall survival curves of two groups of OS patients (low and high BARX1 expression groups) (log-rank test: *p* < 0.0306); actin was used as the loading control

In this study, we differentiated OS and adjacent tissues by HE staining (Fig. 1A). The expression level of BARX1 in the HE-stained tissue samples was detected by IF; the results revealed that BARX1 expression was significantly higher in OS tissue than in adjacent tissue (Fig. 1B). Then, BARX1 mRNA levels were measured in the tumour and adjacent tissues using RT-PCR; the results indicated significant overexpression of BARX1 in the tumour tissue compared with the adjacent tissue (Fig. 1C). These results, in combination with the WB results presented in Fig. 1D, demonstrate that the BARX1 expression level was markedly increased in OS tumour tissue. Kaplan‒Meier analysis showed significantly worse overall survival in patients with high BARX1 expression than in those with low expression (Fig. 1E). In addition, the expression of BARX1 (Tables 2 and 3) was closely related to tumour size, TNM stage, and metastasis.

**Table 2:** BARX1 expression in OS tissues and normal paratumour tissues

Group	*n*	BARX1 expression		*p*
		High (*n*, %)	Low (*n*, %)	
OS tissue	60	36 (60%)	24 (40%)	< 0.001
Peritumoral tissue	60	14 (23.3%)	46 (76.7%)

**Table 3:** The relationship of BARX1 expression with patient clinicopathological features and OS stage

Group	BARX1 expression		*p*
	High (*n*)	Low (*n*)	
Sex			
Male	26	13	0.1151
Female	10	11	
Age (years)			
< 14	22	10	0.139
≥ 14	14	14	
Tumour site			
Tibia/Femur	18	17	0.109
Other	18	7	
Subtype			
Osteoblastoma	13	8	0.825
Other	23	16	
Tumour size (cm)			
< 8	23	9	0.045
≥ 8	13	15	
TNM stage			
I–II	11	17	0.002
III–IV	25	7	
Metastasis			
Yes	30	14	0.032
No	6	10	

### Downregulation of BARX1 expression decreases OS cell proliferation and invasion.

**Fig. 2:**
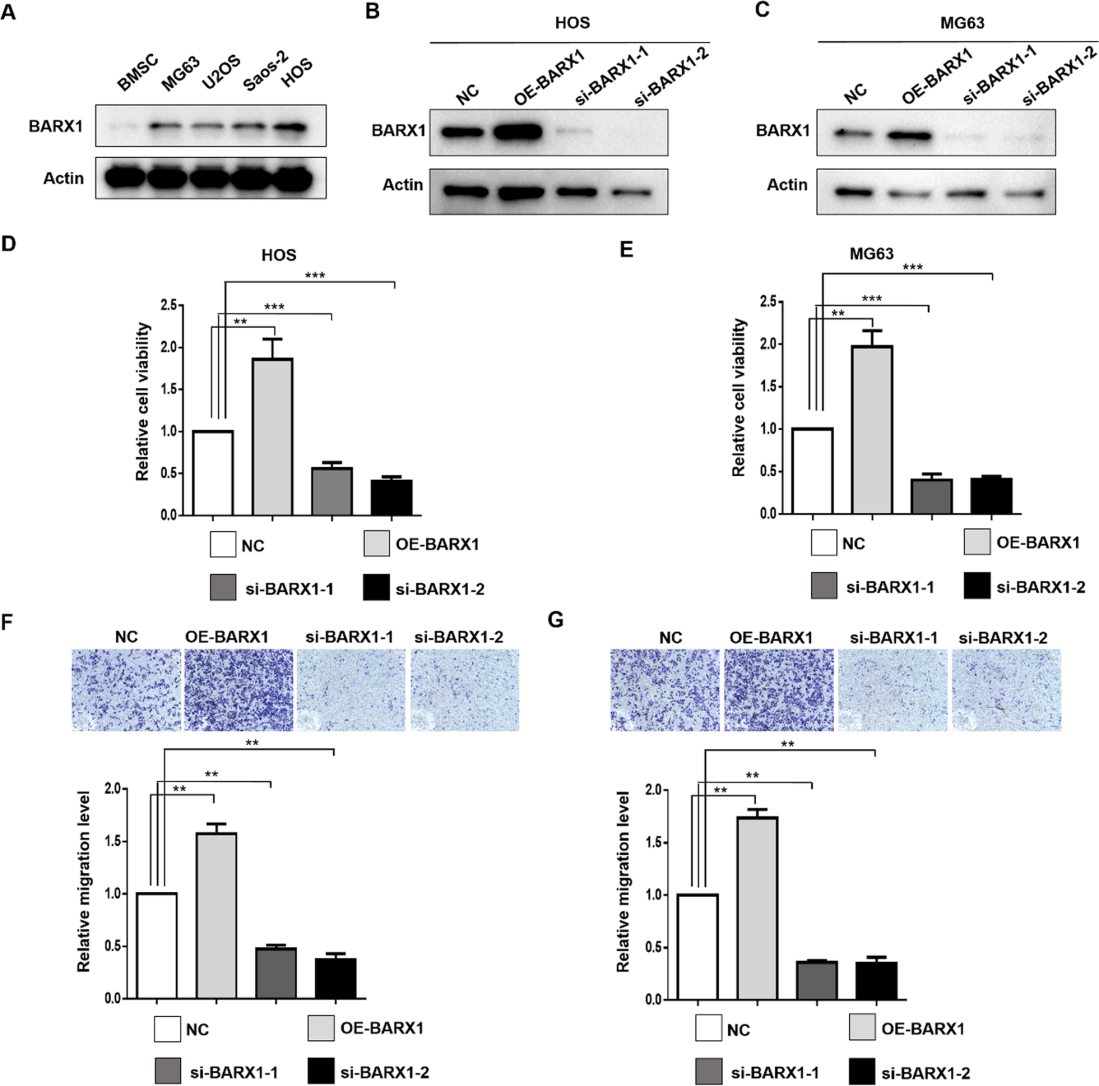
Barx homeobox 1 (BARX1) knockdown decreased OS cell proliferation and invasion. **A** BARX1 expression in four OS cell lines (MG63, U2OS, Saos-2, and HOS) and BMSCs according to Western blotting. **B**/**C** Plasmids or siRNAs were transfected into HOS and MG63 cells, and the efficiency was measured by Western blot assays (**p < 0.01, ***p < 0.001). **D**/**E** After transfection of HOS and MG63 cells with plasmids or siRNA for 48 h, cell growth was assessed via CCK-8 assays. **F**/**G** Plasmids or siRNAs were transfected into HOS and MG63 cells, and Transwell assays were used to assess OS cell invasion

**Fig. 3:**
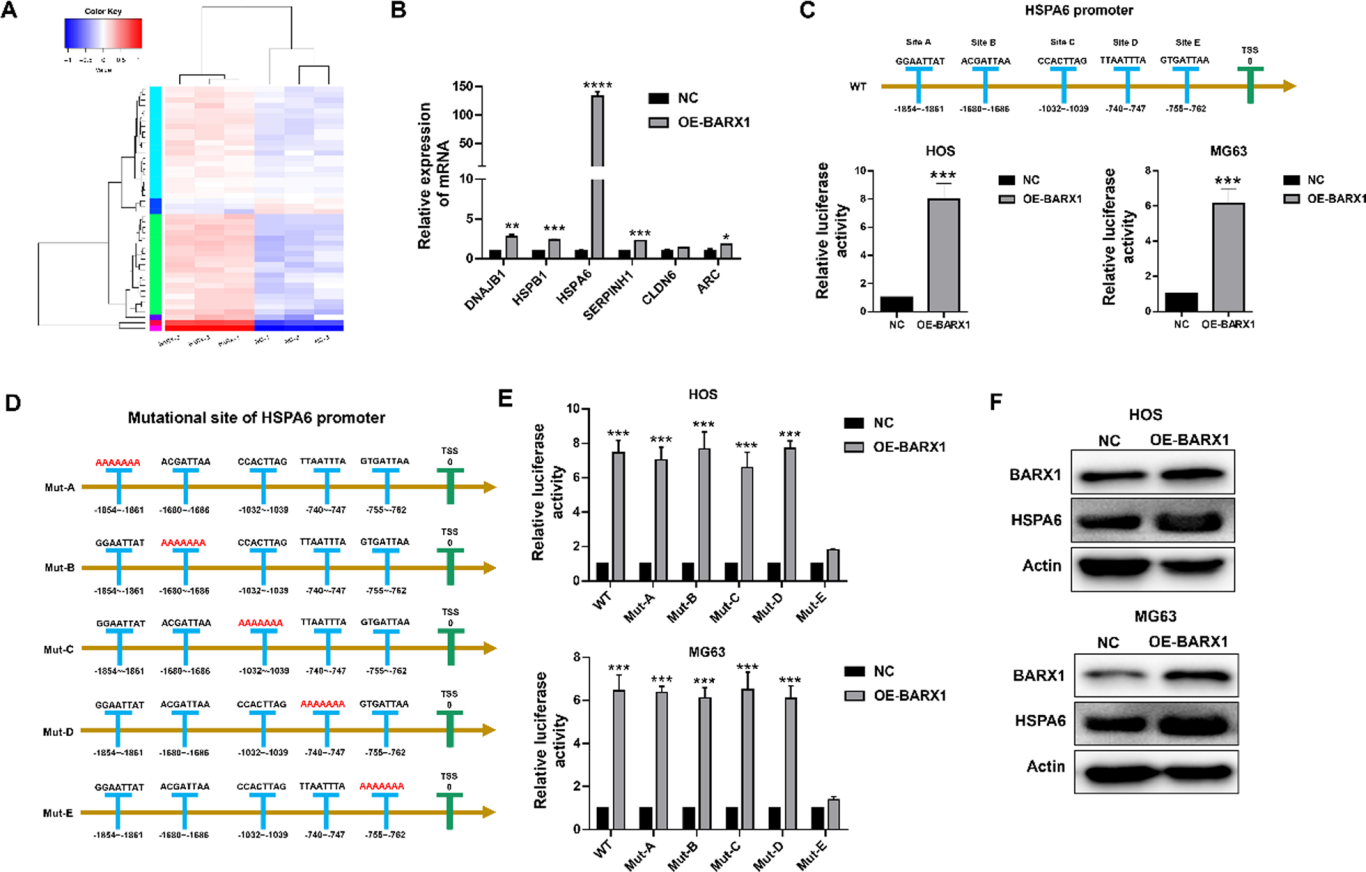
Barx homeobox 1 (BARX1) regulates HSPA6 expression directly in OS cells. **A** RNAs derived from MG63 cells transfected with pcDNA-BARX1 or vectors were extracted and subjected to RNA sequencing. Heatmap of the DEGs in MG63 cells with BARX1 knockdown compared with controls. **B** MG63 cells were transfected with either the overexpression plasmid of BARX1 or the negative control (NC). After 48 h, RNA was extracted, and qPCR was performed to detect the expression of DNAJB1, HSPAB1, HSPA6, SERPINH1, CLDN6, and ARC. **C** The binding sites of BARX1 on the HSPA6 promoter. A dual-luciferase assay was used to analyse the effect of BARX1 on the HSPA6 promoter. **D** Mutation of the predicted binding sites of BARX1 on the HSPA6 promoter. **E** A dual-luciferase assay was used to analyse the effect of BARX1 on the HSPA6 promoter with different mutational sites. **F** HOS and MG63 cells were transfected with the BARX1 overexpression plasmid for 48 h. Western blotting was performed to determine the HSPA6 protein level

To investigate the role of BARX1 in OS cells, BARX1 protein expression was evaluated first in four different OS cell lines (MG63, U2OS, Saos-2, and HOS) using BMSCs as the normal control. As Fig. 2A shows, BARX1 was significantly overexpressed in the four OS cell lines compared with BMSCs. We then amplified or reduced BARX1 expression in HOS and MG63 cells using an overexpression plasmid or siRNA, respectively. The efficiency of the overexpression plasmid and siRNA was identified by WB, as shown in Fig. 2B and C. The transfection efficiency is shown in S Fig. 3. To further define the influence of BARX1 on OS cell proliferation, a CCK-8 assay was conducted to measure cell proliferation after transfection with the overexpression plasmid or siRNA. As Fig. 2D and E shows, the proliferation ability of OS cells approximately doubled after transfection with the overexpression plasmid. In contrast, the downregulation of BARX1 expression by siRNA markedly suppressed OS cell proliferation, indicating that BARX1 is an OS cell proliferation inducer.

To further verify the role of BARX1 in OS cell invasion, a Transwell experiment was conducted. As Fig. 2F and G shows, BARX1 overexpression significantly increased OS cell invasion. However, inhibition of BARX1 expression suppressed OS cell invasion. These data demonstrate that BARX1 acts as an oncogene in OS by promoting OS cell proliferation and invasion.

### BARX1 directly regulates HSPA6 expression in OS cells

BARX1 is defined as a transcription factor [6]. BARX1 was found to be crucial for regulating OS cell proliferation and invasion; hence, to clarify the mechanism underlying that function, RNA-seq was used to identify potential downstream BARX1 targets. As shown in Fig. 3A, pcDNA-BARX1 or vectors were transfected into MG63 cells for RNA-seq. All sample data were subjected to standard quality control prior to difference analysis (Additional file 1: Figure S1). A total of 46 differentially expressed genes (DEGs) (|log twofold-change|> 1, *p* < 0.05) were identified in MG63 cells overexpressing BARX1 compared with the control cells. KEGG pathway and GO enrichment analyses indicated that BARX1 participates in the VEGF pathway and the functional response to unfolded proteins (Additional file 1: Figure S2). These results strongly suggest that BARX1 functions as a tumour inhibitor by suppressing the TNF pathway. Subsequently, we selected the six most upregulated genes for verification. As Fig. 3B shows, HSPA6 was significantly overexpressed after BARX1 overexpression in MG63 cells, suggesting that HSPA6 may be a direct downstream target of BARX1.

To confirm the relationship between BARX1 and HSPA6, we performed bioinformatics analysis using the JASPAR database. As shown in Fig. 3C, there were five sites on the promoter of HSPA6 that were appropriate for BARX1 binding. A dual-luciferase assay was used to determine whether BARX1 could induce HSPA6 promoter activity (Fig. 3C). To accurately identify the target site on the HSPA6 promoter, we constructed five mutants of the HSPA6 promoter (each containing one mutation), mut-A to mut-E (Fig. 3D). Luciferase activity was decreased in the mut-E group, indicating that the E site was the binding site of BARX1 in the HSPA6 promoter (Fig. 3E).

To further validate whether HSPA6 expression was upregulated by BARX1, the HSPA6 expression level in OS cells was detected by WB. As shown in Fig. 3F, HSPA6 expression was increased after BARX1 overexpression in OS cells, demonstrating that HSPA6 is a direct BARX1 target in OS cells.

### BARX1 function depends on HSPA6 expression

**Fig. 4:**
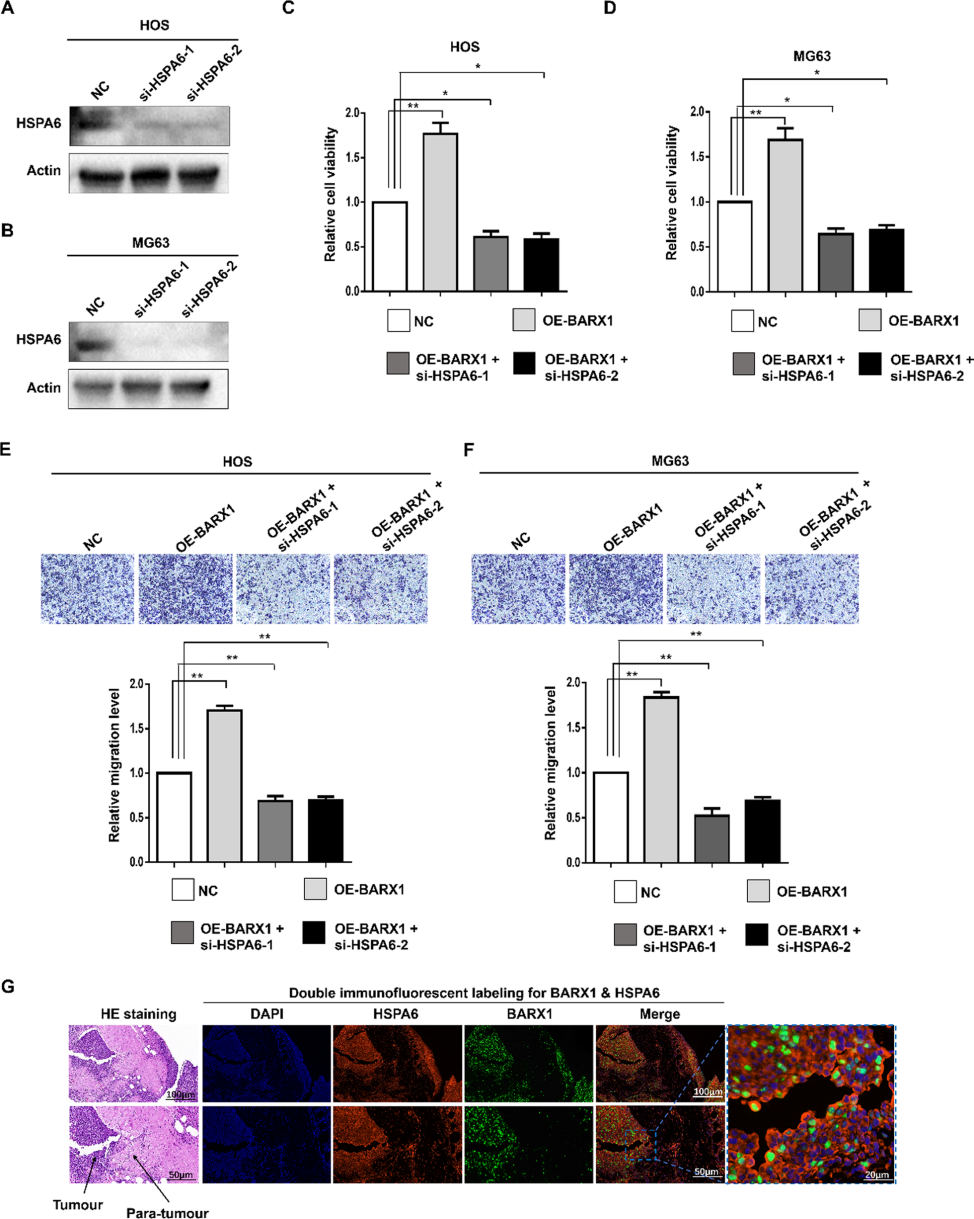
The function of Barx homeobox 1 (BARX1) depends on HSPA6. **A**/**B** Western blotting was performed to determine the efficacy of si-HSPA6. **C**/**D** HOS and MG63 cells were transfected with either BARX1 or si-HSPA6, and a CCK-8 assay was performed to detect cell proliferation. **E**/**F** A Western blot assay was performed on HOS and MG63 cells transfected with BARX1 or si-HSPA6. A Transwell assay was performed to detect cell invasion. **G** A dual immunofluorescence labelling assay was performed to detect the expression of BARX1 and HSPA6 in OS tissue

HSPA6 belongs to the heat shock 70 protein family. Although HSPA6 plays an important role in various tumours, whether HSPA6 can affect the progression of OS remains unclear [13]. To determine whether HSPA6 is involved in BARX1-mediated promotion of OS cell proliferation and invasion, we used siRNA to knockdown HSPA6 in OS cells with enforced BARX1 expression. The efficacy of si-HSPA6 was detected by WB (Fig. 4A and B). Cell growth was assessed by CCK-8 assay. As Fig. 4C and D shows, silencing HSPA6 expression significantly decreased OS cell proliferation induced by BARX1. In addition, HSPA6 depletion inhibited OS cell invasion induced by enforced BARX1 expression (Fig. 4E and F). We further assessed whether BARX1 and HSPA6 could colocalize in OS tissues. As shown in Fig. 4G, the IF assay indicated that BARX1 was overexpressed and associated with HSPA6 overexpression in OS tumour tissues, which revealed the regulatory effect of BARX1 on HSPA6 from a clinical perspective.

## Discussion

OS, which is derived from primitive bone-forming mesenchymal cells, is a common primary bone malignancy [14]. High-grade OS treatment has progressed remarkably since the advent of chemotherapy in the 1970s. Nevertheless, the survival rate remains unsatisfactory because of metastasis and relapse. The biology of OS is complex and not well understood [15], and there are many clinical challenges related to OS, including wide histological heterogeneity, lack of biomarkers, high local aggressiveness, and rapid metastasis.

Increasing evidence shows that BARX1, as a transcription factor, is a two-way regulator of tumour diseases: it can not only promote but also inhibit tumour occurrence and progression [12, 16]. Our study found that the increased expression of BARX1 was associated with a poor prognosis in OS patients and promoted OS cell proliferation and invasion, while downregulation of BARX1 suppressed OS cell proliferation and invasion, indicating that BARX1 acts as an oncogene in OS.

HSPA6 was first identified as an HSP70 family member by Leung et al. in 1990 [17]. Heat shock proteins are overexpressed in different tumours, including breast, prostate, colorectal, and lung cancers, as well as OS [18], where their overexpression is correlated with a poor prognosis and a high rate of chemotherapy resistance [19–21]. High HSP70 levels in many cancers facilitate the survival of these cancer cells [22]. Downregulation of HSP70 strongly reduced tumorigenicity in experimental models [23]. HSP70 also participates in several biological processes that directly enhance tumorigenesis, including cell proliferation, apoptosis, angiogenesis, migration, and drug resistance. In this study, we performed bioinformatics analysis and found that HSPA6 may be the direct target gene of BARX1. HSPA6 can directly or indirectly inhibit or promote tumour occurrence and development. For example, bladder and cervical cancer tumour cell growth, migration, and invasion could be inhibited by increasing the expression of HSPA6 [24]. Knocking down HSPA6 promoted triple-negative breast cancer cell growth, migration, and invasion [25]. Our results revealed the coexpression of BARX1 and HSPA6 in the OS cell line; HSPA6 expression was also high in cells with BARX1 overexpression, and the expression of HSPA6 was not significant in cells with low expression of BARX1. Furthermore, through cell experiments, we found that knocking out HSPA6 while overexpressing BARX1 almost completely abolished the proliferation- and invasion-promoting effects of BARX1. The above results suggest that HSPA6 may be an effector molecule of the BARX1-mediated malignant behaviour of OS.

In summary, this research clinically confirmed the tumour-promoting effect of BARX1 in OS at the cellular and molecular levels and demonstrated that BARX1 enhances OS cell proliferation and invasion by regulating the downstream effector molecule HSPA6, providing an innovative basic theory for studying OS. However, there are still some shortcomings to this study, and an animal model needs to be used to further verify the experimental findings.

The obtained results are helpful for further understanding the biological function of BARX1 and its role in OS; these results provide an in-depth understanding of the OS pathogenesis and recurrence mechanism and provide ideas for OS prognosis evaluation and targeted treatment.

## Supplementary Information

**Additional file 1.** **Supplementary Figure 1.** RNA sequencing gene expression analysis and advanced QC. **Supplementary Figure 2.** RNA sequencing GO analysis and KEGG pathway analysis.

## Data Availability

The datasets generated or analysed during the current study are available from the corresponding author on reasonable request.

## Abbreviations

OS: Osteosarcoma
BARX1: Barx homeobox 1
IF: Immunofluorescence
BMSCs: Bone marrow mesenchymal stem cells
HSPA6: Heat shock 70-kDa protein 6
NSCLC: Non-small cell lung cancer
HCC: Hepatocellular carcinoma
IF: Immunofluorescence
