# Supplementary material for: BARX1 promotes osteosarcoma cell proliferation and invasion by regulating HSPA6 expression
Source: J Orthop Surg Res. 2023 Mar 16;18:211. doi: 10.1186/s13018-023-03690-z (PMC10018937; doi:10.1186/s13018-023-03690-z)
Supplement: Supplementary file 1 — Additional file 1. Supplementary Figure 1. RNA sequencing gene expression analysis and advanced QC. Supplementary Figure 2. RNA sequencing GO analysis and KEGG pathway analysis. [file 13018_2023_3690_MOESM1_ESM.docx]

**Supplementary Material**

**
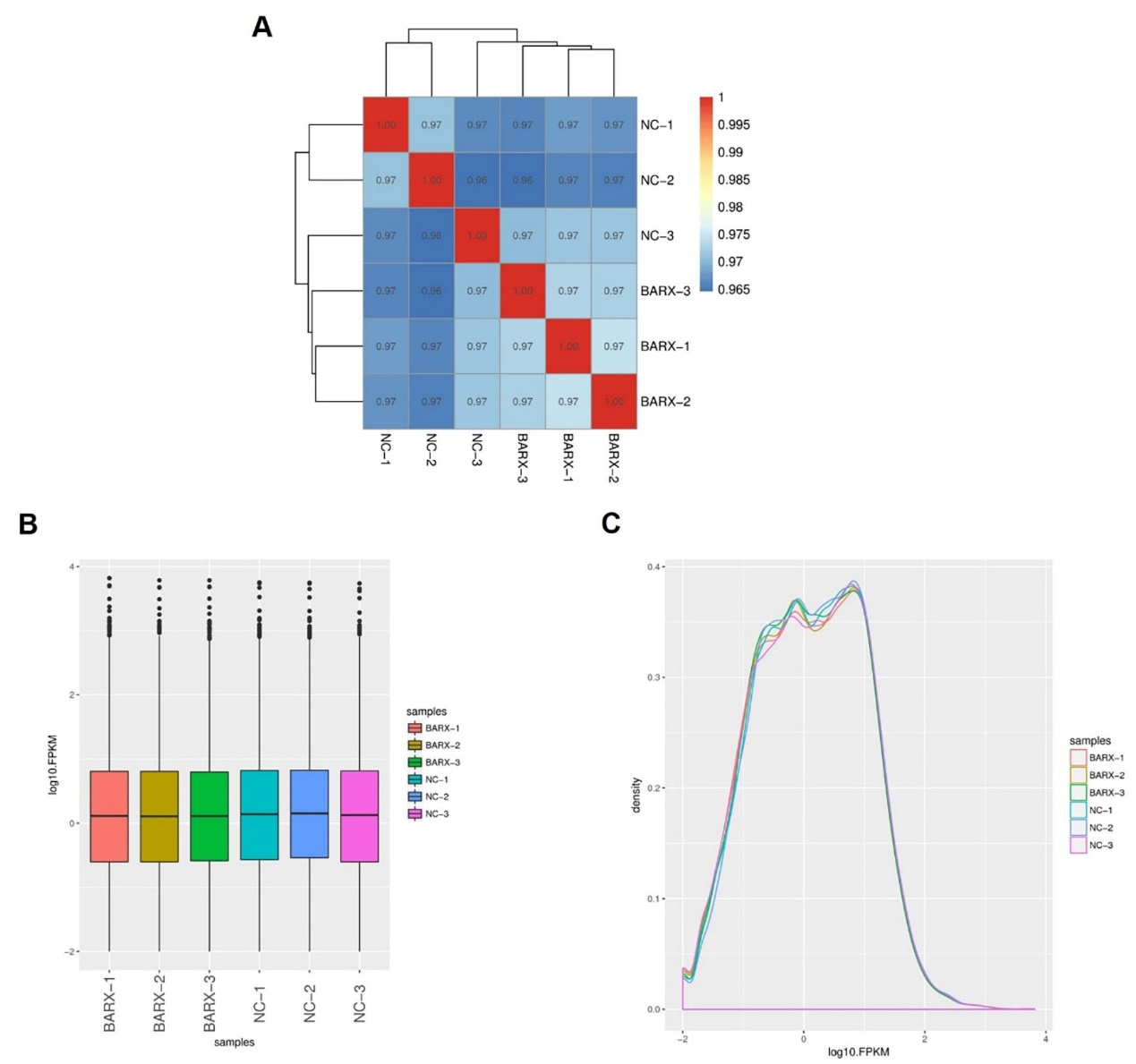
**

Supplementary Figure 1. RNA sequencing gene expression analysis and advanced QC; A. Heatmap of NC and BARX1-OE samples; B-C. Fragments per kilobase of exon per million mapped reads or boxplot distribution in NC and BARX1-OE samples.


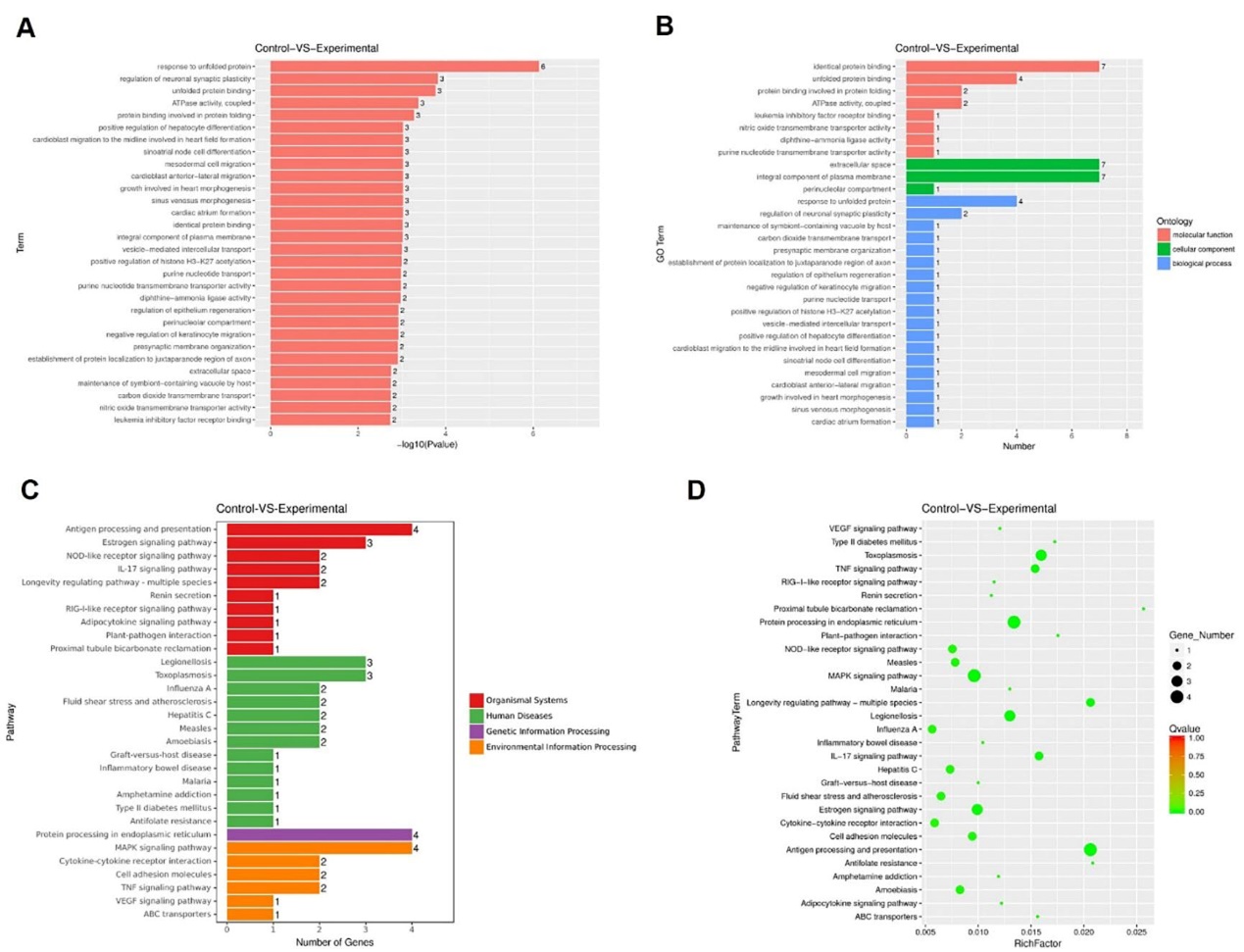


Supplementary Figure 2. A-B GO analysis of the mRNA sequencing results; C-D KEGG pathway analysis of the mRNA sequencing results.

**Note:** All data are reported as the mean ± standard deviation (* P < 0.05; ** P < 0.01; *** P < 0.001; **** P < 0.0001). The experiments were replicated three times.
